# Shift Work and Circadian Dysregulation of Reproduction

**DOI:** 10.3389/fendo.2013.00092

**Published:** 2013-08-07

**Authors:** Karen L. Gamble, David Resuehr, Carl Hirschie Johnson

**Affiliations:** ^1^Department of Psychiatry and Behavioral Neurobiology, University of Alabama at Birmingham, Birmingham, AL, USA; ^2^Department of Cell and Developmental and Integrative Biology, University of Alabama at Birmingham, Birmingham, AL, USA; ^3^Department of Biological Sciences, Vanderbilt University, Nashville, TN, USA

**Keywords:** endocrinology, pregnancy failure, misalignment, sleep, circadian disruption, infertility

## Abstract

Health impairments, including reproductive issues, are associated with working nights or rotating shifts. For example, shift work has been associated with an increased risk of irregular menstrual cycles, endometriosis, infertility, miscarriage, low birth weight or pre-term delivery, and reduced incidence of breastfeeding. Based on what is known about circadian regulation of endocrine rhythms in rodents (and much less in humans), the circadian clock is an integral regulatory part of the reproductive system. When this 24-h program is disordered by environmental perturbation (such as shift work) or genetic alterations, the endocrine system can be impaired. The purpose of this review is to explore the hypothesis that misalignment of reproductive hormones with the environmental light-dark cycle and/or sleep-wake rhythms can disrupt menstrual cycles, pregnancy, and parturition. We highlight the role of the circadian clock in regulating human reproductive physiology and shift work-induced pathology within each step of the reproductive axis while exploring potential mechanisms from the animal model literature. In addition to documenting the reproductive hazards of shift work, we also point out important gaps in our knowledge as critical areas for future investigation. For example, future studies should examine whether forced desynchronization disrupts gonadotropin secretion rhythms and whether there are sleep/wake schedules that are better or worse for the adaptation of the reproductive system to shift work. These studies are necessary in order to define not only whether or not shift work-induced circadian misalignment impairs reproductive capacity, but also to identify strategies for the future that can minimize this desynchronization.

## Introduction

The National Sleep Foundation 2008 poll estimates that ∼7% of American workers are shift workers (1), and the U.S. Bureau of Labor Statistics estimates that nearly 15% of the workforce is employed on an alternative work schedule such as evening, night, or rotating shift work (2). Shift work is associated with an increased risk of developing cardiovascular/metabolic/gastrointestinal disorders, some types of cancer, and mental disorders including depression and anxiety (3–5). In women, shift workers have a higher incidence of obesity and high blood pressure (6), breast cancer (7), and reproductive issues (8–10). For example, shift work has been associated with an increased risk of irregular menstrual cycles (11), endometriosis (12), miscarriage (13), low birth weight or pre-term delivery (14, 15), and reduced incidence of breastfeeding (16). The purpose of this review is to highlight the role of circadian clock in regulating human reproductive physiology and shift work-induced pathology within each step of the reproductive axis.

The circadian clock allows organisms to anticipate regular and daily repeating events that occur at approximately the same time of day, and this anticipation enables upregulation of key physiological pathways so that an appropriate physiological or behavioral response can be elicited at the correct time. In mammals, these 24-h rhythms in physiology are orchestrated by a primary clock in the suprachiasmatic nucleus (SCN) of the hypothalamus. The SCN coordinates other central circadian oscillators (e.g., the hypothalamus and pituitary gland) that drive rhythms in peripheral tissues (17), including endocrine tissues such as the adrenal gland, thyroid gland, adipocytes, pancreas, and gonads (18). (For an overview of basic human reproductive endocrinology, see Box 1). Within both the central clocks in the brain and peripheral oscillators, the 24-h timing mechanism appears to be the same and is composed of a set of genes (clock genes) and respective proteins that form a feedback loop [for review see (19)] which rhythmically regulates many output genes (clock-controlled genes or ccg’s) as detailed in Box 2. For the purpose of this review, we will focus on the circadian clock genes *CLOCK*, *NPAS2*, *BMAL1* (a.k.a. *ARNTL*), *PER1*, and *PER2*.

Box 1 **Basic reproductive physiology overview.**

Female mammals exhibit a cyclical flux of hormones controlling follicular maturation and ovulation. The approximately 28-day menstrual cycles of women are tightly orchestrated by the HPG axis, which coordinates peripheral organs with the central nervous system (CNS). The primary signal from the CNS is GnRH, which is secreted in short pulses averaging once every 90 min. GnRH also stimulates the anterior pituitary to release FSH and LH, which promote follicular development. Estradiol is produced by developing follicles in the ovaries; its synthesis is a carefully regulated system that is coordinated by feedback mechanisms between the hypothalamus, anterior pituitary, and ovaries. The release of estradiol from developing ovarian follicles stimulates proliferation of the uterine endometrium and negatively regulates the further release of GnRH and FSH. One follicle outgrows all of the others which undergo atresia. As estradiol concentrations peak, they trigger the surge release of GnRH into the hypophyseal portal blood system. This GnRH surge triggers a surge of LH which acts on the ovary and induces ovulation. The follicle releases the ovum as it ruptures and undergoes luteinization. During this, the secretory cycle stage, large amounts of progesterone and estradiol are secreted by the corpus luteum. The secretory phase lasts for 10–16 days in women. During this phase the endometrium becomes increasingly thicker as endometrial glands and blood vessels become increasingly tortuous in preparation for implantation of a fertilized ovum. Simultaneously, estrogen in particular and progesterone to a lesser extent negatively feed back to the anterior pituitary and maintain low secretory rates of LH and FSH. In the absence of pregnancy, involution of the corpus luteum usually occurs after the 12th day. The sudden cessation of its inhibitory effect on the anterior pituitary allows for the renewed release of LH and FSH to begin a new ovarian cycle. At the same time, paucity of secretion of progesterone and estradiol lead to the breakdown of endometrial lining and menstruation.

Box 2 **The molecular circadian clock.**

**(A)** The 24-h timing of the molecular clock is orchestrated by a set of clock genes and proteins that form a positive feedback loop when CLOCK dimerizes with BMAL1 and binds to the E-box elements of *PER* and *CRY* genes, activating transcription. Negative control of transcription occurs when *PER/CRY* dimerize, and this complex translocates into the nucleus where it inhibits the transcription of its own genes, through inhibition of *BMAL1* and *CLOCK* transcription.

Circadian clock-gene expression is rhythmic in peripheral tissues, including in human PBMCs (49–52) with *hPER1/2* peaking at the sleep-to-wake transition and *hBMAL1* peaking at the end of the wake period (53). **(B)** Circadian clock-gene expression has been reported in a majority of neuroendocrine tissues (86). The neural timing of the SCN is believed to signal to neuroendocrine cells (e.g., hypothalamic GnRH neurons) which in turn (via GnRH) drive the rhythmic secretion of pituitary LH and subsequent ovulation. Rhythms of clock-gene expression have in fact been reported in many brain regions involved in controlling the HPG axis, such as the hypothalamus and the pituitary gland, the latter being the earliest and best described circadian oscillator in the HPG axis (21, 87, 88). Several peripheral mammalian reproductive tissues in both male [e.g., extra-testicular ducts (89)] and female [e.g., ovary, uterus, and oviducts (45, 90–94)] rodents have also been shown to exhibit 24-h oscillations in circadian clock-gene expression and circadian clock-controlled gene expression rhythms [for review, see (23)]. For example, in the ovary, the transcriptional rhythms of the circadian clock genes *per1/2* and *bmal1* peak around light offset and onset, respectively, regardless of ovarian cycle stage (8, 23).

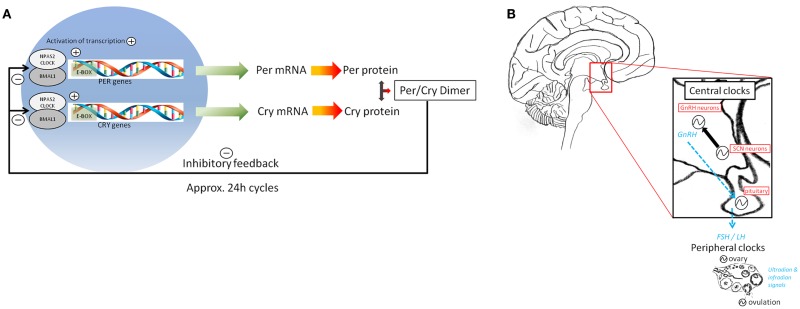

The circadian rhythms of clock-gene expression observed in reproductive brain areas suggest that this neural timing system drives and orchestrates neuroendocrine events ultimately leading up to the pre-ovulatory luteinizing hormone (LH) surge and ovulation [reviewed by (18)]. The SCN has been shown to be necessary for normal functioning of the hypothalamic pituitary gonadal (HPG) axis, and rhythms of clock-gene expression have been recorded in brain regions controlling both the HPG and hypothalamic-pituitary-adrenal (HPA) axes. The earliest and best described circadian oscillator in the HPG axis is the pituitary gland (18). Here, rhythmic gene expression of prolactin and gonadotropin releasing hormone (GnRH) receptors has been shown to be mediated via clock-gene regulatory elements [i.e., E-Boxes; (20, 21)]. Furthermore, GnRH secreted by the hypothalamus appears to activate clock-gene expression in pituitary gonadotrophs through the same intracellular mechanism used to drive LH gene expression (22). In the more distally located ovarian clock, CLOCK and BMAL1 transcriptionally regulate a clock-controlled gene whose protein product is a key transcription factor (COX2) involved in prostaglandin synthesis (23). This growing body of work linking circadian and reproductive systems supports the notion that the coordinated timing of circadian clocks in reproductive brain areas such as the hypothalamus, forebrain, and pituitary and more peripheral circadian oscillators in the gonads may not only facilitate but be a prerequisite for establishment and maintenance of reproductive health.

Endogenous rhythmicity must be entrained or synchronized to the environment, and this process primarily occurs through photic input to the SCN from the retina. Specifically, environmental light is detected in intrinsically photoreceptive, melanopsin-containing retinal ganglion cells, which transmit this information directly to the SCN via the dedicated retinohypothalamic tract (24). Through a multi-synaptic pathway, the SCN drives 24-h rhythms in the production of the pineal hormone melatonin and is also critical for acute, light-induced suppression of melatonin synthesis at night (25, 26). Other environmental stimuli including exercise and feeding behavior can also entrain circadian rhythmicity. For example, when rodents consume food 12 h out of phase with activity rhythms, the rhythmic clock-gene expression in the SCN remains the same while clock-gene expression shifts in the liver, kidney, heart, and pancreas, leading to a state of internal desynchronization (27, 28). In addition, transient internal desynchronization induced by a 6-h delay of the light-dark cycle in rats alters sleep architecture (29). In fact, even a simple 6-h shift in the light-dark cycle takes 6–12 days for clock-gene expression rhythms to completely adjust, with different peripheral tissues requiring varying amounts of time to shift (30, 31). Detailed investigation of the effects of a 6 h advance of the light-dark cycle on ovarian rhythmic phase has revealed that the ovarian clock is not fully shifted by 6 days, but phase synchrony is fully restored by 12 days. Moreover, endocrine signals from the pituitary [LH and follicle stimulating hormone (FSH)] are capable of resetting the ovarian clock phase (31). In addition to transient desynchronization, chronic internal desynchronization using repeated, weekly 6 h advances in the light-dark cycle in rodents, can result in greatly increased mortality in aged or immune-challenged animals (32, 33). Given that shift workers commonly prefer to shift back to night-sleep on days-off (34), a better understanding of the internal desynchronization of endocrine tissues induced by frequent shifts in sleep/wake behavior is critically important to finding solutions to the reproductive health hazards of shift work.

## Circadian Regulation of Fertility

### Circadian clock regulation in endocrinology and in reproductive tissues

In the 1960s, Aschoff elegantly demonstrated that body temperature, urine excretion, and sleep/wake patterns in humans exhibit a circadian rhythm (i.e., persistence of time-of-day-dependent rhythms when environmental cues remain constant) (35). Since that time, numerous studies have also reported circadian regulation of plasma cortisol levels in humans, including the persistence of a ∼24-h rhythm even when participants maintain a 28-h behavioral day, inducing desynchrony of circadian and sleep/wake behavioral cycles. It is interesting to note that despite the primary clock rhythm being ∼24 h (truly circadian), many endocrine rhythms underlying its regulation are either ultradian (shorter than 24 h; e.g., 90-min GnRH pulses) or infradian (longer than 24 h; e.g., 28-day LH surge). In any case, these endogenous, central, clock-controlled rhythms are important because previous studies have shown that peak cortisol levels in the morning (cortisol awakening response) correlate with the timing of the LH surge, which typically peaks at the end of the sleep episode (36, 37) consistent with rodent models [see (38) for review]. Indeed, an intact central clock in the SCN has been shown to be necessary for the LH surge and subsequent ovulation (39). In addition to LH, serum concentrations of FSH, estrogen or 17β-estradiol (E2), and progesterone (P4) show significant time-of-day variation (37, 40) and increase shortly after awakening specifically in women with regular menstrual cycles in the estradiol (E2) dependent proliferative phase and progesterone (P4) dependent secretory phase (41). However, one recent study found that estradiol, LH, FSH, and glycoprotein free α-subunit did not exhibit a circadian rhythm in young, healthy, normal cycling women in the proliferative phase living on a constant routine in the absence of environmental time cues (42). Given the vast amount of literature on circadian regulation of endocrine rhythms in rodents (8, 10, 18), it is surprising how little is known about circadian regulation of human endocrine rhythms, as noted in (8). It will be important for future circadian rhythm studies to distinguish between effects of the circadian clock and sleep on endocrine rhythmicity because partial sleep deprivation can modulate thyroid stimulating hormone, prolactin, LH, and estradiol (43).

A hint that the circadian molecular clock is regulating time-of-day-dependent variation in the reproductive hormones that affect fertility are the recent findings that a polymorphism in the circadian clock-gene *ARNTL* (rs2278749) confers increased risk of miscarriages, and a polymorphism (rs11673746) in the circadian clock-gene *NPAS2* (a CLOCK homolog) may be protective and is associated with decreased incidence of miscarriage (44). These studies in humans are consistent with compelling results in mice that global knockout of the *Bmal1* gene (the only single gene knockout that results in behavioral rhythmicity) causes infertility in both males and females. Male knockout mice were also characterized by low testosterone, high LH, and low FSH, as well as small testes and low sperm count (45).

### Circadian clock desynchrony disrupts endocrinology

In humans, internal desynchronization can be induced by a forced 28-h sleep-wake cycle (8-h sleep, 20-h awake) which is outside the range of entrainment for the human circadian clock. Rhythms of some metabolites such as leptin and glucose predominately follow the 28-h behavioral cycle, while cortisol rhythms follow the 24-h circadian cycle (5), consistent with the idea that the cortisol rhythm is driven by the primary circadian clock in the SCN. After four cycles, this protocol results in circadian misalignment, in which the behavioral sleep-wake cycle is 12-h out of phase with the circadian cycle. In these misaligned conditions, leptin rhythms are blunted, postprandial glucose and insulin are increased, and cortisol rhythms are 180° out of phase with the behavioral rhythm. Nearly half of the participants undergoing the 28-h cycle exhibited a pre-diabetic state during circadian misalignment. In field studies of shift work, the phase of the melatonin rhythm in night shift workers exhibits large inter-individual variability even when measured after the last night shift worked in permanent night shifters (46) or after 12 days of night shift (47). In one report, cortisol rhythms took five consecutive shifts to adapt to the new behavioral sleep phase, and even then, 25% of the workers rhythms never adapted (48). Circadian clock-gene expression is rhythmic in human peripheral blood mononuclear cells (PBMCs) (49–52) with *hPER1/2* peaking at the sleep-to-wake transition and *hBMAL1* peaking at the end of the wake period (53). After 9 days of simulated shift work (forced day-sleep), *hPER1/2* rhythms from PBMCs are fully shifted while *hBMAL1* rhythms are not (54). However, it is important to note that after only 3 days of simulated shift work, *hPER1* and *hBMAL1* expression rhythms are not fully adjusted (54). Although circadian desynchronization of central and peripheral clocks in the reproductive system has yet to be demonstrated (an important area of future research), the above studies suggest that it is possible that misalignment of reproductive system timing also occurs during shift work. If so, this misalignment may contribute to reproductive dysfunction.

**Figure 1:**
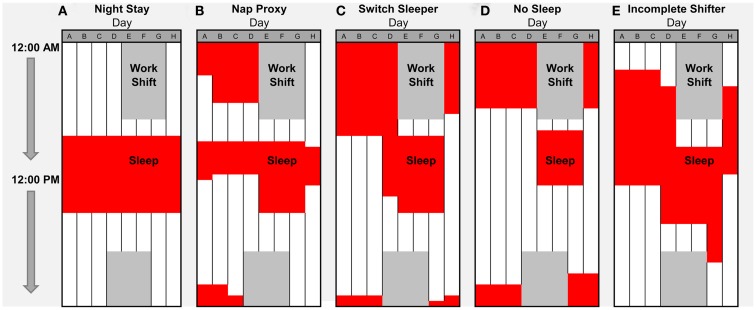
**Self-selected sleep strategies for night shift work weeks in nurses**. We recently characterized off-shift behavioral sleep strategies of full-time, primarily European–American nurses at Vanderbilt University Medical Center (VUMC) who completed a self-report survey (34), including a typical work-week schedule for night shift at Vanderbilt Hospital for which nurses indicated typical sleep times. **(A–E)** Five representative strategies for days-off (and percentage among night shift workers) were: **(A)** Night Stay (2.4%); **(B)** Nap Proxy (12.2%); **(C)** Switch Sleeper (49.2%); **(D)** No Sleep (24.3%); and **(E)** Incomplete Switcher (11.9%). Red indicates sleep times and dark gray indicates night shift schedule from 7:00 p.m. to 7:00 a.m. on Days D–G. Surprisingly, ∼1 out of 4 night shift nurses chose to switch between days and nights via a>24-h sleep deprivation period (the No Sleep strategy).

Circadian clock disruption can have deleterious effects on reproduction. Although a common single nucleotide polymorphism in the *CLOCK* gene (rs1801260) was not significantly associated with endometriosis diagnosis, working night shift greatly increases the risk of this disease. Moreover, women who changed their sleep patterns on days-off were particularly vulnerable (12). This finding is especially compelling for nurses in light of our recent finding that ∼97% of nurses choose to switch their sleeping patterns to some form of nocturnal sleep on days-off (34), as depicted and described in Figure 1. Surprisingly, ∼1 out of 4 night shift nurses chose to switch between days and nights via a>24-h sleep deprivation period (the No Sleep strategy). In this sample, sleep deprivation commonly occurred just before the first work day, which could impair performance and alertness on the job. Nurses adopting the No Sleep strategy indicated the lowest adaptation levels (in terms of sleep routine regularity and fatigue) to their current shift schedule of 7:00 p.m. to 7:00 a.m. Interestingly, routinely switching sleep/wake patterns may also contribute to irregular menstrual cycles, as noted in several studies (55, 56). Specifically, a survey study of reproductive issues in hospital nurses showed that over half of the nurses working nights or rotating shifts complain of painful and irregular menstrual cycles, including changes in the cycle length, menstrual flow, dysmenorrhea, or duration of menstrual bleeding. In addition, a significantly larger percentage of Taiwanese hospital nurses with fixed night shift schedules report menstrual cycles of less than 25 days (57). Likewise, rotating hospital nurses are more likely to have irregular menstrual cycles than permanent day shift nurses (58). In a previous cohort, this same group of researchers found that rotating shift nurses reported variability in menstrual cycle length (some of which were over 33 days). Since lengthening of the menstrual cycle is associated with the length of the follicular phase, these results suggest rotating shift work may induce a delay in ovulation (59). This result is consistent with the classical study in rodent models, in which prevention of the LH surge during the critical circadian window will delay estrus until the same time on the following day (60). More recently, data from the very large cross-sectional Nurses Health Study indicated that the increased length of rotating shift work is significantly associated with a higher relative risk of irregular, extremely long, or extremely short menstrual cycles, after adjustment for contributing covariates (11). Unfortunately, this study was not designed to distinguish rotating shift work from night shift work. Altogether, these results in shift workers are consistent with the interpretation that environmental disruption of the circadian clock has deleterious effects on women’s reproductive health.

There are some data suggesting that the circadian-regulated pineal hormone melatonin has an antagonistic relationship with the gonadal hormones. Given that melatonin is critical for signaling seasonal day length and the onset of winter-like inhibition of reproductive function in most photoperiodic animals, it is not entirely surprising that melatonin can be inversely related to estrogen levels (61). Specifically, melatonin can interact with gonadal rhythms as evidenced by (i) melatonin-induced augmentation of the LH surge and (ii) reduced ovarian activity in short day-length environments characterized by increased nocturnal melatonin (62). Finally, a clinical trial of melatonin therapy in perimenopausal and menopausal women with low, medium, and high nocturnal melatonin showed that administration of melatonin reduced LH and FSH of women with initial low melatonin (63). This apparent antagonistic relationship between melatonin and gonadotropins, taken together with the correlated cortisol awakening response and LH surge, suggest that misalignment of cortisol and melatonin rhythms during shift work (46, 47) could be an underlying mechanism for impaired fertility and dysregulated menstrual cycles.

## Circadian Regulation of Pregnancy

### Circadian clock control of pregnancy hormones

During the third trimester, pregnant women normally exhibit significant daily variation in plasma adrenocorticotropic hormone (ACTH), cortisol, and progesterone, but not corticotrophin releasing hormone (CRH) or estradiol, as reported by Magiakou et al. (64). These authors suggested that during late pregnancy, mild hypercortisolism (although still rhythmic) results from a placental CRH positive feedback loop. Circadian regulation of the HPA axis during pregnancy is consistent with circadian regulation of the clock-controlled plasminogen activator inhibitor 1 (important for embryonic development), uterine circadian clock-gene regulation, and maternal placental circadian clock-gene regulation in rodent models (8). In addition to the role of the circadian clock in regulating the HPA axis during pregnancy, the clock may also play an important role in maintaining blood pressure homeostasis during pregnancy. Specifically, timed administration of low doses of acetylsalicylic acid (aspirin) in a double blind, randomized clinical trial has been successful in treating pregnant women with a high risk of developing pre-eclampsia (65). Specifically, women who were given daily doses of acetylsalicylic acid 8 h after waking or just before bed time were less likely to develop pre-eclampsia/gestational hypertension or to have a pre-term delivery than those who took daily doses immediately after waking. In addition, the later time of administration significantly and reversibly reduced blood pressure during pregnancy. Importantly, mothers with afternoon/evening treatment with acetylsalicylic acid gave birth to infants with significantly greater gestational age and higher birth weight. Taken together, these studies suggest that circadian regulation of HPA axis function and blood pressure are important factors during pregnancy.

### Shift work-induced circadian desynchrony during pregnancy

Several studies have examined the impact of shift work on maternal health and pregnancy outcomes. In general, shift work (especially rotating work or irregular hours) during pregnancy is associated with pre-term birth, low birth weight, and higher risk of spontaneous abortion or miscarriage (66). Although one previous study noted that night shift work does not affect urinary estradiol levels of pregnant hospital nurses (67, 68), this within-subjects study of nurses on a rapid rotation shift had too few subjects to be statistically persuasive, and a more recent meta-analysis that examined a large number of subjects found that the pooled risk estimates for pre-term delivery, low birth weight, and small for gestational age births increased with shift work [(15) but see also (14)]. In addition, examination of factory shift workers revealed that persistent rotating shifts led to lower birth weights and those infants in the bottom 20th percentile of birth weight were more likely to be born from mothers who work persistent rotating shifts (69). Despite the clear time-of-day dependency of treating pre-eclampsia noted above (65), a very large epidemiology study of workers in Taiwan (>24,000) found that working evening or rotating shifts did not increase the risk of gestational hypertension and pre-eclampsia above the risk for non-working mothers (70).

In rodent models, the impact of circadian desynchrony on pregnancy outcome is quite evident from the work of several studies. For example, an early study found that pregnant mice housed on a 22- or 26-h day (which is outside of the range of entrainment for this species) results in a higher incidence of embryo resorption and poor pup survival (71). In addition, repeated 6-h phase advances or delays (a chronic jet lag model) in mice resulted in a significant decrease in the number of full-term pregnancies resulting from successful matings (72). A body of work in rodents has shown an important role for circadian regulation of prolactin in maintaining pregnancy [reviewed in (38)]. Mice with mutations in the *Clock* gene have insufficient prolactin release, suggesting that disruption of the circadian clock could impact pregnancy outcome through dysregulation of prolactin (38). Finally, circadian dysregulation during pregnancy may not only affect the health of the mother, but may also affect the health of the pup even after maturity. For example, rats that underwent chronic jet lag (12-h shifts) during pregnancy produced pups with metabolic problems such as obesity, hyperleptinemia, and modulation of glucose tolerance/insulin insensitivity (73).

## Circadian Regulation of Parturition

### Circadian/diurnal regulation of labor/delivery

Over the years, many studies have noted that either the onset of labor or birth occurs more often at certain times of the day than others. For example, the onset of labor and spontaneous membrane rupture peaks at night between midnight and 4:00 a.m. (74–76). Several factors such as bacterial inflammation of fetal membranes (chorioamnionitis), time between membrane rupture and labor onset, as well as primiparous versus multiparous mothers, can modulate the timing of labor or membrane rupture. For example, if labor occurs within 3 h of spontaneous membrane rupture, labor is more likely to begin earlier in the day (75). In addition, labor onset in first pregnancies is more likely to occur either in the early evening (i.e., 8:00 p.m.) or early morning (i.e., 8:00 a.m.), and this time-of-day labor onset bimodal rhythm appears to be absent in multiparous pregnancies (77). In contrast to labor onset timing, which is most likely to begin during the night, the timing of births peak during the day around 1:00–2:00 p.m. for primiparous births and slightly earlier for multiparous births (i.e., early morning) (78). Finally, there may be seasonal rhythms – for example, one study noted that births are more common from September to November compared to the winter months (December to February) (78).

Given these temporal differences in parturition, it is not surprising that several studies have found that some interventions during labor are more successful at certain times of the day than others. For example, one study conducted a nested, randomized, controlled clinical trial comparing morning (8:00 a.m.) versus evening (8:00 p.m.) administration of prostaglandin and its success rate in inducing labor. While there were no differences in whether or not birth occurred within 24 h of induction or whether or not a cesarean delivery was used, morning inductions required less oxytocin, had a shorter induction to birth interval, and were less likely to result in instrumental vaginal births for women in their first pregnancies (79). However, it is important to note that a recent meta-analysis of perinatal mortality found no overall difference between morning and evening prostaglandin application or oxytocin-delivery in either maternal or neonatal outcomes (80). The day/night profile of melatonin secretion is one putative underlying mechanism for time-of-day differences in labor and delivery or in the success rate of labor intervention. A recent review highlights this potential role for melatonin, noting that melatonin and oxytocin work in concert to induce contraction of smooth muscle myometrial cells in the uterus (81).

### Circadian dysfunction and labor/delivery

In people, sleep and circadian disruption induced by shift work can affect labor and pregnancy outcome. For example, severely disrupted sleep during late pregnancy is more likely to result in cesarean delivery, and even sleep restriction to less than 6] per night is sufficient to increase the risk of long labor and cesarean delivery. Interestingly, fatigue during labor and delivery is not an underlying factor (82). In addition, pregnant Chinese textile workers who work rotating shifts are more likely to give birth at younger gestational ages and to infants with a low birth weight (83), consistent with the Swedish Midwife Study, which found that night work is significantly associated with pre-term birth and small for gestational age births (84). Overall, this link between shift work and pregnancy outcome is consistent with findings in mice with genetic knockout of the *Clock* gene. Only 57% of these mutant mice deliver viable pups and the rest either reabsorb their pups or go into a long, extended labor that does not end in delivery (85). Finally, even after birth, shift work can impact new moms’ ability to breastfeed. One study found that breastfeeding rates among night shift workers are reduced at 1 month and 2 months after birth (16).

## Conclusion and Suggestions

**Figure 2:**
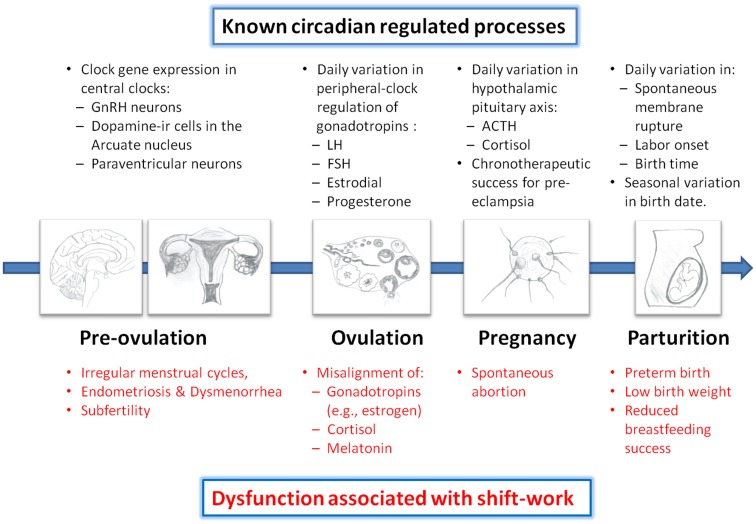
**Circadian regulation of reproduction and impairment associated with shift work**. Evidence suggests that the circadian clock regulates each part of the reproductive axis from timing of neuronal activity in hypothalamic neurons to the day-night variation in the release of pregnancy hormones. Dysregulation of circadian rhythms, as often occurs with shift work, results in increased risk of adverse consequences at each step of the reproductive pathway. See text for references. GnRH, gonadotropin releasing hormone; LH, luteinizing hormone; FSH, follicle stimulating hormone; ACTH, adrenocorticotropic hormone.

In summary, there is a growing amount of evidence supporting the notion that the circadian clock is involved in regulation of nearly every part of the reproductive pathway. For example, circadian clock-gene expression has been localized to neuroendocrine centers in the brain (e.g., hypothalamic GnRH neurons and pituitary cells) and reproductive tissues (e.g., ovary, fallopian tube, uterus), and shown to be necessary for the proper regulation of prolactin, gonadotropins (e.g., LH and FSH), and the GnRH-receptor. Moreover, day-night variation in blood pressure during pregnancy, and timing of labor and/or birth suggest the involvement of the circadian clock. Thus, it is conceivable that perturbations of the circadian system misalign hormones and gonadotropins during the reproductive cycle in a shift work environment. In turn, this can lead to interference with menstrual cycles, pregnancy, and parturition, resulting in increased risk of infertility, spontaneous abortions, pre-term births, low birth weights, and difficulty breastfeeding (Figure 2). While documenting these hazards of shift work is an important ongoing mission, it is now imperative for future studies to empirically show how shift work creates circadian misalignment of gonadotropins and other endocrine hormones in humans. The ultimate goal is to determine how to reduce this misalignment and the consequential pathologies. Given that many shift workers revert to nocturnal sleep patterns on days-off (34), entrainment of endocrine rhythms to night shift is not likely a viable solution. Attention to off-shift behavioral sleep strategies and determination of which strategies produce the least amount of misalignment of circadian clock-controlled rhythms (including reproductive hormones) could lead to potential solutions to the reproductive hazards of shift work. For example, in our study of nurses on night shift, we identified five different sleep/wake schedules that nurses had themselves selected (described in Figure 1). Based on self-reported adaptation and performance, we suggest that strategies such as “Incomplete Shifter” or “Switch Sleeper” be implemented to minimize the loss of performance due to sleep deprivation. Tests of these sleep/wake strategies for their impact on the reproductive system could identify schedules that minimize the shift work-induced disruptions in the reproductive axis reviewed in this paper.

## Conflict of Interest Statement

The authors declare that the research was conducted in the absence of any commercial or financial relationships that could be construed as a potential conflict of interest.
